# Involvement of males in antenatal care, birth preparedness, exclusive breast feeding and immunizations for children in Kathmandu, Nepal

**DOI:** 10.1186/1471-2393-13-14

**Published:** 2013-01-16

**Authors:** Dharma Nand Bhatta

**Affiliations:** 1 Department of Public Health, Pokhara University, Nobel College, Sinamangal, Kathmandu, Nepal

**Keywords:** Male involvement, Nepal, ANC, Birth preparedness, Exclusive breast feeding, Immunization

## Abstract

**Background:**

Men in patriarchal societies of developing countries are often identified as decision makers in all aspects of day-to-day life. The study explores the factors associated with male involvement in ANC, birth plans, exclusive breastfeeding and immunization of children.

**Methods:**

A cross-sectional survey was conducted among 2178 married males between May and December 2010 in Kathmandu, Nepal. Twenty one clusters were selected for data collection using structured questionnaire. Adjusted odds ratios (AORs) and 95% confidence intervals (95% CI) of associated factors were estimated by stepwise backward likelihood ratio method.

**Results:**

This study determined the percentage of males who accompanied their partners for ANC (39.3%), arranged SBA (47.9%) and accompanied them for child immunization (10.9%). Factors found associated with respect to different activities were as follows: accompanied their partners on antenatal visits – uneducated or primary level education (AOR, 5.68, 95% CI, 4.44-7.27), income NPR 5001 (1USD = 85 NPR) or above (1.47, 1.20-1.80) and aged above 25 years (2.51, 1.89-3.33); arranged money for delivery – uneducated or primary level education (7.34, 5.84-9.23), income NPR 5001 or above (1.80, 1.48-2.20) and aged above 25 years (1.55, 1.18-2.03); arranged SBA – uneducated or primary level education (17.14, 12.65-23.22) and income NPR 5001 or above (2.89, 2.36-3.54); arranged transportation – uneducated or primary level education (17.65, 11.84-26.32), income NPR 5001 or above (1.69, 1.40-2.04) and aged above 25 years (1.69, 1.27-2.24); encouraged exclusive breast feeding – uneducated or primary level education (5.48, 4.39-6.83) and aged above 25 years (1.35, 1.03-1.77); accompanied their partners for immunization their children – uneducated or primary level education (3.88, 2.53-5.96) and aged above 25 years (1.72, 1.11-2.64).

**Conclusions:**

Men who were uneducated or had primary level education, aged above 25 years, had higher income, formal employment, came from Hindu religion and non-indigenous ethnicities demonstrated greater involvement and these factors should be emphatically considered during maternal health program development.

## Background

In this study male involvement is defined as men attending antenatal care (ANC) visits, birth plans, encouraging exclusive breast feeding and immunization for their children. Men not only act decision-makers for women and children’s access to health services, but also through abuse or neglect, men’s actions can have a direct bearing on the health of their female partners and children [1]. Thus, it has become imperative to foster women’s empowerment. Men are often identified as the sole decision-makers in all aspects [2]. In 1997, the United Nations Population Fund (UNFPA) described an agenda for the *International Conference on Population and Development*, Cairo and *Fourth World Conference on Women*, Beijing, in which men would play a proactive role in the empowerment of women [3].

Maternal deaths arise from pregnancy, childbirth or postpartum complications, but their occurrences could be reduced by making birth plans for pregnant women, their partners and their families. Antenatal care, birth plans and complication-readiness are crucial for timely access to skilled maternal and neonatal services. These factors promote active preparation and decision-making for delivery [4]. The key elements of the birth plan includes recognition of danger signs, a plan for skilled birth attendant, place of delivery, and arrange money for transport or other costs [5]. The birth plan is a very important strategy in developing countries, where obstetric services are weak and thus contribute significantly to maternal and neonatal morbidity and mortality [6]. A husband at antenatal clinic is rare in many communities and it is unthinkable to find men accompanying with their partners during ANC and delivery [7].

However, men hold social and economic power and have tremendous control over their partners, especially in developing countries. They decide the timing and conditions of sexual relations, family size, and whether or not their spouses will utilize available health care services [8]. Strategies for involving men in maternal health services should aim at raising their awareness about emergency obstetric conditions, and engaging them in birth plans and complication readiness [5]. Male involvement enables men to support their spouses to utilize obstetric services and the couple would adequately prepare for birth complications. This would lead to a reduction in all three phases of delay: delay in the decision to seek care; delay in reaching care; and finally, delay in receiving care. The male partner can play a crucial role especially in the first and second phases of delay in developing countries and thereby positively impact birth outcomes [9].

The main objective of this study is to determine the associated factors affecting male involvement in ANC, birth plans, exclusive breast feeding and immunization. Nepal has culturally dynamic and patriarchic societies. The low status of women, socio-cultural barriers to seeking care: women’s mobility, ability to command resources, decision-making abilities, beliefs and practices surrounding childbirth and delivery has great impact on women’s health. Most of the reproductive health programs fail to address these factors in Nepal. Enhancement of male involvement is necessary in culturally dynamic societies like Nepal to improve the women’s health and reduce maternal morbidity and mortality.

## Methods

A cross-sectional study was carried out in different twenty village development committees (VDCs) and one municipality of Kathmandu district which is the central part of Nepal. The study was conducted between May and December 2010. The study inclusion criteria were: a married male of a household head whose wife had given birth to at least one child, and his willingness to participate in the study.

A two-staged cluster sampling technique was used. The clusters were selected in the first stage and respondents were selected in the second stage. A total of twenty village development committees (VDCs) and one municipality were randomly selected from the Kathmandu district. A cluster was a group of households in the same geographical area. In this study a cluster consisted of between 50 and 300 households. Firstly the wards for each selected village development committees and municipality were listed in ascending order. Where wards had more than 400 households, those wards were divided into sub wards. If wards had less than 50 households, they were merged with neighboring wards to make one cluster with between 50 and 300 households. One ward from each VDCs and municipality were selected as a cluster. Twenty one clusters were selected using probability proportionate to the number of households. To reach sampled household heads in 21 clusters, 100 household heads from each cluster of VDCs and 200 household heads from municipality were taken for interview as a sample. Among the total 2200 household heads, 22 were refused to take participation in the study. Hence the total sample size was estimated to be 2178 men. Household heads were interviewed within the cluster using interval sampling. Interview was started from the central part of the cluster and household heads selected randomly.

A structured, interviewer-administered questionnaire were used to collect the data on i) socio-demographic variables such as age, education level, income, ethnicity, religion and employment status, which were the predictors; ii) participation in birth process, such as accompanied with wives on ANC visit; iii) birth plans, based on the number of arrangements the male had made, including money saved for delivery, arranged transportation, arranged skilled birth attendance (SBA); and iv) encouraged exclusive breast feeding and accompanied for child immunization. The primary end point was male involvement in ANC, birth plans, exclusive breast feeding and immunization.

Data was analyzed to provide frequencies and percentages for categorical variables and means and standard deviations for numerical variables. Univariate and multivariate logistic regression (stepwise backward likelihood ratio method) was conducted to analyze factors that were associated with male involvement in ANC, birth plans, immunization, breast feeding, while assessing for interaction and collinearity. In both the univariate and multivariate analyses, P value of less than 0.05 was considered as significant. The Hosmer and Lemeshow test was used to assess “goodness of fit” of the models and the likelihood ratio test to assess the relative contribution of terms entered into the model. P-value of less than 0.05 was considered to add the variables in the equations in the process of stepwise model. Religion with accompanied for ANC; ethnicity, religion and employment with accompanied for immunization were removed in the adjusted analysis. The data was summarized and the odds ratios (ORs) estimated; and their corresponding 95% confidence intervals (95% CI) were computed. Ethical clearance was provided by the institutional review committee of State University of Bangladesh (approval no. 12/09/010) and Nepal Health Research Council. Ethical guidelines were followed and participants were recruited after getting informed written consent. The data was cleaned and cross-checked daily before and after data entry for completeness and accuracy. The analysis was done in SPSS 16.0 version.

## Results

Table 1 shows socio-demographic and economic characteristics of the study group. The mean ± SD of age was 33.24±8.92 years, and 85.5% of men were 25 years and above. Among the total respondents, 69.4% of men had secondary or higher level of education, 47.5% of men had formal employment, 87.2% of men were Hindu, and 81.4% of men were non-indigenous ethnicities. As shown in Table 2, 855 (39.3%) of men accompanied their partners to antenatal visit, while 56.2% of men helped in domestic chores. Only 26.9% of men had knowledge about danger signs in pregnancy, 53.7% of men arranged money for delivery, 47.9% of men arranged SBA, 30.2% of men arranged transportation, and 58.7% of men encouraged their partners for exclusive breast feeding. Majority (95.0%) of men were completed immunization of their child aged under nine month, whereas only 10.9% of men accompanied their partners for their child immunization.

**Table 1:** Socio-demographic characteristics (N=2178)

**Characteristic**	**Frequencies (Percentage)**
**Age** Mean ± standard deviation	33.24±8.92
25 years and above	1863 (85.5)
24 years and young	315 (14.5)
**Income Level** Monthly (NPR)*	
NPR 5001 and above	1098 (50.4)
NPR 5000 and below	1080 (49.6)
**Education Level**	
Secondary or Higher Level	1512 (69.4)
Non or Primary Level	666 (30.6)
**Occupation**	
Formal employee	1035 (47.5)
Informal employee	1143 (52.5)
**Religion**	
Hindu	1899 (87.2)
Other than Hindu	279 (12.8)
**Ethnicity**	
Non-Indigenous	1773 (81.4)
Indigenous	405 (18.6)

*1 USD = 85 NPR.

**Table 2:** characteristics of male involvement (N=2178)

**Characteristic**	**Frequencies (%)**
Accompany with wife for antenatal care (ANC)	855 (39.3)
Agree with mother need more food during pregnancy	2097 (96.3)
Help to wife in domestic chores	1224 (56.2)
Known about danger sign	585 (26.9)
Saving money for delivery	1170 (53.7)
Arranged skill birth attendant for delivery (SBA)	1044 (47.9)
Arranged transportation for delivery	657 (30.2)
Agree with necessity of breast feeding	2169 (99.6)
Encourage for exclusive breast feeding	1278 (58.7)
Immunized child aged under 9 month	2070 (95.0)
Accompany for Immunization (N=2070)	225 (10.9)

Among the 1323 respondents, men said that the reasons for not to accompanied their partners on antenatal visit were: the feeling that this is a woman’s duty (53.0%), they were preoccupied with other tasks (29.3%), they were embarrassed (15.0%), and they had a lack of knowledge (2.7%) (table not shown).

Table 3 shows that the factors associated with men who accompanied their partners on ANC visit. Those men who were uneducated or had primary level education (adjusted odds ratio 5.68, 95% confidence interval 4.44 to 7.27), were aged 25 years or above (2.51, 1.89 to 3.33), with formal employment (1.23, 1.01 to 1.49), had income NPR 5001 (exchange rate 1USD = 85 NPR) or above per month (1.47, 1.20 to 1.80) and of non-indigenous ethnicities (1.53, 1.18 to 1.99) were more likely to accompanied their partners on antenatal visit than men who were educated at secondary or higher level, were aged 24 years or below, had informal or no employment, had income NPR 5000 or less per month and came from indigenous ethnicities.

**Table 3:** Factors associated with accompany for ANC (N = 2178)

**Characteristic**	**Crude Odds ratios with 95% confidence interval**	**Adjusted Odds ratios with 95% confidence interval**
Age above 25 years Age 24 years & young- Ref	2.05 (1.57-2.69)*	2.51 (1.89-3.33)*^a^
Education non or primary Education secondary or higher - Ref	5.73 (4.52-7.25)*	5.68 (4.44-7.27)*^a^
Formal employment Informal or no employment - Ref	1.73 (1.45-2.06)*	1.23 (1.01-1.49)**^a^
^Non-Indigenous ethnicity Indigenous ethnicity - Ref	2.00 (1.58-2.54)*	1.53 (1.18-1.99)*^a^
Income 5001 NPR or above/month 5000 NPR or less/month- Ref	1.89 (1.59-2.25)*	1.47 (1.20-1.80)*^b^

*P<0.001, **P<0.05 (^Non indigenous –*Brahmin & Kshatriya* caste in Nepal).

^a^adjusted with education age, employment, and ethnicity, ^b^adjusted with ethnicity, age, employment, and income.

Men who were uneducated or had primary level education (7.34, 5.84-9.23), were aged 25 years or above (1.55, 1.18-2.03), with formal employment (2.02, 1.66-2.45), had income NPR 5001 or above per month (1.80, 1.48 to 2.20) and of the Hindu religion (3.20, 2.19-4.67) were more likely to arranged money for delivery than men who were educated at secondary or higher level, were aged 24 years or below, had informal or no employment, had income NPR 5000 or less per month and came from other than Hindu religion.

Those men who were uneducated or had primary level education (17.14, 12.65 to 23.22), had income level NPR 5001 or above per month (2.89, 2.36 to 3.54), had formal employment (1.46, 1.19 to 1.78), were non-indigenous ethnicities (1.96, 1.54 to 2.49), and followed the Hindu religion (2.36, 1.70 to 3.28) were more likely to arranged skilled birth attendant (SBA) for delivery than men who were educated at secondary or higher level, had income NPR 5000 or less per month, had informal or no employment, came from indigenous ethnicities and other than Hindu religion.

Men who were uneducated or had primary level education (17.65, 11.84 to 26.32), were aged 25 years or above (1.69, 1.27 to 2.24), Hindu religion (2.04, 1.36 to 3.08), had income NPR 5001 or above per month (1.69, 1.40 to 2.04), had formal employment (1.35, 1.12 to 1.63) and came from non-indigenous ethnicities (1.99, 1.44 to 2.75) were more likely to arranged transportation than men who were educated at secondary or higher level, were aged 24 years or below, came from other than Hindu religion, had income NPR 5000 or less per month, had informal or no employment and came from indigenous ethnicities. (Table 4).

**Table 4:** Factors associated with arrange money for delivery (N = 2178)

**Characteristic**	**Crude Odds ratios with 95% confidence interval**	**Adjusted Odds ratios with 95% confidence interval**
Age above 25 years Age 24 years & young - Ref	1.11 (0.88-1.41)	1.55 (1.18-2.03)*^a^
Education non or primary Education secondary or higher - Ref	8.53 (6.86-10.61)*	7.34 (5.84-9.23)*^a^
Formal employment Informal or no employment - Ref	3.04 (2.55-3.62)*	2.02 (1.66-2.45)*^a^
Hindu religion Other than Hindu religion - Ref	3.29 (2.50-4.32)*	3.20 (2.19-4.67)*^a^
Non-Indigenous ethnicity Indigenous ethnicity - Ref	1.58 (1.27-1.96)*	0.70 (0.51-0.97)**^a^
Income 5001 NPR or above/month 5000 NPR or less/month- Ref	2.67 (2.24-3.17)*	1.80 (1.48-2.20)*^b^
**Factors associated with arrange SBA for delivery (N = 2178)**		
Education non or primary Education secondary or higher - Ref	21.49 (15.96-28.95)*	17.14 (12.65-23.22)*^c^
Hindu religion Other than Hindu religion - Ref	3.01 (2.27-4.00)*	2.36 (1.70-3.28)*^c^
Age above 25 years Age 24 years & young - Ref	0.85 (0.67-1.08)	0.61 (0.47-0.80)*^d^
Income 5001 NPR or above/month 5000 NPR or less/month- Ref	3.30 (2.77-3.94)*	2.89 (2.36-3.54)*^d^
Formal employment Informal or no employment - Ref	2.38 (2.00-2.83)*	1.46 (1.19-1.78)*^d^
Non-Indigenous ethnicity Indigenous ethnicity - Ref	1.87 (1.49-2.34)*	1.96 (1.54-2.49)*^d^
**Factors associated with arrange transportation for delivery (N = 2178)**		
Age above 25 years Age 24 years & young - Ref	1.29 (0.99-1.70)	1.69 (1.27-2.24)*^e^
Education non or primary Education secondary or higher - Ref	16.90 (11.35-25.18)*	17.65 (11.84-26.32)*^e^
Hindu religion Other than Hindu religion - Ref	3.28 (2.28-4.71)*	2.04 (1.36-3.08)*^f^
Non-Indigenous ethnicity Indigenous ethnicity - Ref	2.73 (2.05-3.64)*	1.99 (1.44-2.75)*^f^
Income 5001 NPR or above/month 5000 NPR or less/month- Ref	1.77 (1.47-2.13)*	1.69 (1.40-2.04)*^f^
Formal employment Informal or no employment - Ref	1.40 (1.17-1.69)*	1.35 (1.12-1.63)**^g^

*P<0.001, **P<0.05.

^a^adjusted with education, age, employment, religion and ethnicity, ^b^adjusted with religion, employment, and income.

^c^adjusted with religion, income and education, ^d^adjusted with income, age, employment and ethnicity.

^e^adjusted with education and age, ^f^adjusted with income, religion and ethnicity, ^g^adjusted with ethnicity, employment and religion.

Table 5 shows that the factors associated with males who encouraged exclusive breast feeding. Men with an income of NPR 5001 or above per month (0.70, 0.58 to 0.87), and of Hindu religion (0.50, 0.36 to 0.69) were less likely to encouraged exclusive breast feeding than men with an income NPR 5000 or less per month, and followed other than Hindu religion. Those men who were uneducated or had primary level education (5.48, 4.39 to 6.83), were aged 25 years or above (1.35, 1.03 to 1.77), had formal employment (1.32, 1.10 to 1.57) and of non-indigenous ethnicities (1.82, 1.37 to 2.41) were more likely to encouraged exclusive breast feeding than men who were educated at secondary or higher level, were aged 24 years or below, had informal or no employment and came from indigenous ethnicities.

**Table 5:** Factors associated with encourage exclusive breast feeding (N = 2178)

**Characteristic**	**Crude Odds ratios with 95% confidence interval**	**Adjusted Odds ratios with 95% confidence interval**
Age above 25 years Age 24 years & young - Ref	1.08 (0.85-1.37)	1.35 (1.03-1.77)**^a^
Education non or primary Education secondary or higher - Ref	4.50 (3.70-5.46)*	5.48 (4.39-6.83)*^a^
Income 5001 NPR or above/month 5000 NPR or less/month- Ref	1.26 (1.06-1.50)**	0.70 (0.58-0.87)*^a^
Hindu religion Other than Hindu religion - Ref	1.03 (0.80-1.33)	0.50 (0.36-0.69)*^a^
Non-Indigenous ethnicity Indigenous ethnicity - Ref	1.63 (1.31-2.02)*	1.82 (1.37-2.41)*^a^
Formal employment Informal or no employment - Ref	1.27 (1.07-1.51)**	1.32 (1.10-1.57)**^b^
**Factors associated with accompany for Immunization (N = 2070)**		
Characteristic	Crude Odds ratios with 95% confidence interval	Adjusted Odds ratios with 95% confidence interval
Age above 25 years Age 24 years & young - Ref	1.36 (0.89-2.07)	1.72 (1.11-2.64)**^c^
Education non or primary Education secondary or higher - Ref	3.11 (2.05-4.70)*	3.88 (2.53-5.96)*^c^
Income 5001 NPR or above/month 5000 NPR or less/month- Ref	0.81 (0.62-1.07)	0.57 (0.43-0.77)*^c^

*P<0.001, **P<0.05.

^a^adjusted with education, age, income, religion and ethnicity, ^b^adjusted with religion, employment, and ethnicity, ^c^adjusted with education, age and income.

Additionally, those men who were uneducated or had primary level education (3.88, 2.53 to 5.96) and were aged 25 years or above (1.72, 1.11 to 2.64) were more likely to accompanied their partners for immunization of children than those men who were educated at secondary or higher level and were aged 24 years or below. Men with an income of NPR 5001 or above per month (0.57, 0.43 to 0.77) were less likely to accompanied their partners for the immunization of their child.

## Discussion

This study examined the factors associated with male involvement in ANC, birth plans, exclusive breast feeding and immunization. The findings highlighted the associated factors such as education, age, employment, income, ethnicity and religion. Literature shows that the couples in which male had a higher level of education were better informed and so were likely to involved on birth plan, and were more socially or financially empowered to make the necessary decisions [4,5,7]. This study revealed that the men with higher age, uneducated or had primary level education, had higher income, had formal employment and came from non-indigenous ethnicities were found associated factors for males involvement on ANC visit. Men who were knowledgeable and obtained health education were more likely to accompany their spouses for ANC visit [4,5,10]. This study highlighted that the reasons said by males for not to accompany their partners on ANC visit were: the belief that it is a woman’s duty, being preoccupied with work, and a feeling of embarrassment. In addition, cultural diversities might affect a change in this attitude. This study finding shows that 39.3% of males accompanied their partners for ANC visits. Previous findings suggested that providing information to male partners about attending antenatal care might increase their involvement, as well as greater preparedness in the case of pregnancy [8-13]. Unfortunately, according to most studies, male partner involvement in maternal and child health is still low in many countries. Antenatal care represents a window of opportunity for information; education, and communication of, and with, pregnant women so that they will make appropriate choices during pregnancy, especially when they are in danger. However, this opportunity is often missed [14,15] and compounded by different associated factors [16].

Literature shows that 61% of pregnant women had adequate preparations for delivery, while only 4.8% had preparation for emergency complications [17]. Other study shows that 62.9% of men arranged money for delivery, 67% of men knew at least one danger sign in pregnancy, while only 6.9% knew of three or more danger signs [18]. In this study only 26.9% of men had knowledge about least danger signs and 53.7% of men arranged money for delivery. Men with higher age, uneducated or had primary level education, had higher income, had formal employment and came with Hindu religion were found associated factors for higher involvement with arranged money for delivery. Previous study shows that 84.3% of men arranged transportation to hospital for delivery [18]. However, in this study 30.2% of men arranged transportation to hospital for delivery and men with higher age, uneducated or had primary level education, had higher income, had formal employment, came from Hindu religion and non-indigenous ethnicities demonstrated higher involvement and arranged transportation. Men who were uneducated or had primary level education, had higher income, had formal employment, with non-indigenous ethnicity and Hindu religion demonstrated higher involvement and arranged of SBA. Nearly half of the males arranged SBA for delivery. Previous study finding shows that several men were actively involved in birth plans and complication readiness when their spouses were pregnant or in labor [19].

Previous study results suggested that the pregnant women and their male partners should be given health education together, as this would result in a greater net impact on maternal health behaviors, compared to educating the women alone [20]. However, in this study, 58.7% of men encouraged their partners for exclusive breast feeding where men with higher age, uneducated or had primary level education, had formal employment and non-indigenous ethnicities were found associated factors for higher involvement. Only 10.3% of men accompanied their partners for immunizations of their child and men who were with higher age and uneducated or had primary level education demonstrated greater involvement. Literature shows that women’s ability to seek health care or implement it is often determined by the household head, who usually is the husband [21,22]. This study explored the involvement of men in reproductive health and demonstrates how its importance in positively impacting maternal and child health.

### Strengths and weaknesses of the study

The main strength of this research lies in its large sample size, sampling strategy for data collection, and the fact that the clusters were taken from the same geographical location. A set of reliability and validation rules were applied and all associated factors were taken after indication of significance in the “goodness of fit” for the models.

In assessment, this study also had several limitations: This study was community-based among male respondents, such that results were not generalizable to the hospital services. Secondly, a few questions were inquired into after delivery, which may create some bias. There is no way one can verify that the responses were not the socially desirable responses, more so considering that the interviews were conducted within the community. The interviews were conducted confidentially, in the absence of spouses, to avoid biased responses.

## Conclusions

Men who were uneducated or had primary level education, were aged above 25 years, had higher income, formal employment, came from Hindu religion and non-indigenous ethnicities demonstrated greater involvement and these factors should be emphatically considered during maternal health program development.

## Competing interests

The author declares no conflict of interest.

## Pre-publication history

The pre-publication history for this paper can be accessed here:

http://www.biomedcentral.com/1471-2393/13/14/prepub
